# Crystal structure, Hirshfeld surface analysis and DFT studies of (*E*)-4-methyl-2-{[(4-methyl­phen­yl)imino]­meth­yl}phenol

**DOI:** 10.1107/S2056989020007847

**Published:** 2020-06-16

**Authors:** Nermin Kahveci Yagci, Md. Serajul Haque Faizi, Alev Sema Aydin, Necmi Dege, Onur Erman Dogan, Erbil Agar, Ashraf Mashrai

**Affiliations:** a Kirikkale University, Faculty of Arts and Sciences, Physics Department, 71450 Kirikkale, Turkey; b Department of Chemistry, Langat Singh College, B.R.A. Bihar University, Muzaffarpur, Bihar-842001, India; c Department of Chemistry, Faculty of Arts and Sciences, Ondokuz Mayıs University, Samsun, 55200, Turkey; d Department of Physics, Faculty of Arts and Sciences, Ondokuz Mayıs University, Samsun, 55200, Turkey; e Faculty of Pharmacy, University of Science and Technology, Ibb Branch, Ibb, Yemen

**Keywords:** crystal structure, Schiff base, intra­molecular hydrogen bonding, 2-hy­droxy-5-methyl­benzaldehyde, *p*-tolyl­amine

## Abstract

In the title compound, C_15_H_15_NO, the configuration of the C=N bond of the Schiff base is *E*, and an intra­molecular O—H⋯N hydrogen bond is observed, forming an intra­molecular *S*(6) ring motif. The phenol ring is inclined by 45.73 (2)° from the plane of the aniline ring. In the crystal, mol­ecules are linked along the *b* axis by O—H⋯N and C—H⋯O hydrogen bonds, forming polymeric chains. The Hirshfeld surface analysis of the crystal structure indicates that the most important contributions for the packing arrangement are from H⋯H (56.9%) and H⋯C/C⋯H (31.2%) inter­actions. The density functional theory (DFT) optimized structure at the B3LYP/ 6–311 G(d,p) level is compared with the experimentally determined mol­ecular structure, and the HOMO–LUMO energy gap is provided. The crystal studied was refined as an inversion twin.

## Abstract 2

The title compound, C_15_H_15_NO, shows an intra­molecular O—H⋯N hydrogen bond and the aromatic rings are tilted by 45.73 (2)°.

## Chemical context   

Azomethines (known as Schiff bases), having imine groups (CH=N) and benzyl rings alternately in the main chain and being conjugated, are inter­esting materials for a wide spectrum of applications, in particular as metal-ion complexing agents and in biological systems (Hökelek *et al.*, 2004 ▸; Moroz *et al.*, 2012 ▸; Kansız & Dege, 2018 ▸). Schiff bases are important in various areas of chemistry and biochemistry because of their biological activity (El-masry *et al.*, 2000 ▸) and photochromic properties. They also have applications in various fields such as the measurement and control of radiation intensities in imaging systems and optical computers (Elmalı *et al.*, 1999 ▸), and electronics, optoelectronics and photonics (Iwan *et al.*, 2007 ▸). They are used as anion sensors (Dalapati *et al.*, 2011 ▸) and as non-linear optics compounds (Sun *et al.*, 2012 ▸). The present work is part of an ongoing structural study of Schiff bases and their utilization in the synthesis of new organic, excited-state proton-transfer compounds, and fluorescent chemosensors (Faizi *et al.*, 2016 ▸, 2018 ▸; Kumar *et al.*, 2018 ▸; Mukherjee *et al.*, 2018 ▸). We report herein the crystal structure as well as the Hirshfeld surface analysis of the title Schiff base (*E*)-4-methyl-2-{[(4-methyl­phen­yl)imino]­meth­yl}phenol, (I). A comparison between the calculated structure [obtained using density functional theory at the B3LYP/6-311 G(d,p) level] and the experimental data is also presented.
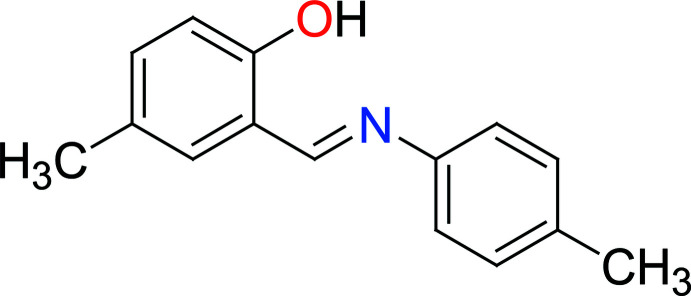


## Structural commentary   

**Figure 1:**
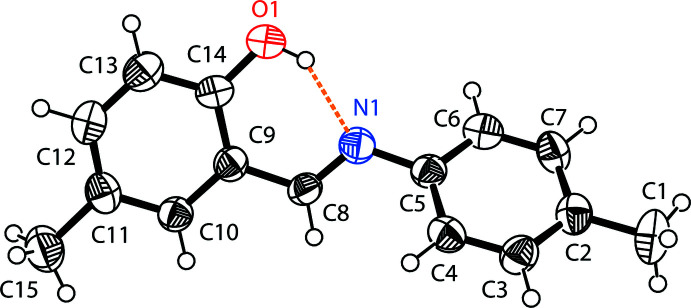
The mol­ecular structure of (I), with the atom-labelling scheme. Displacement ellipsoids are drawn at the 40% probability level. The intra­molecular O—H⋯N hydrogen bond (see Table 1 ▸) is shown as a dashed line.

The mol­ecular structure of the title compound is illustrated in Fig. 1 ▸. An intra­molecular O—H⋯N hydrogen bond is observed, which forms an *S*(6) ring motif (Table 1 ▸ and Fig. 1 ▸). This is a relatively common feature in analogous imine–phenol compounds (see *Database survey* section). The imine group, which displays a C9—C8—N1—C5 torsion angle of −169.8 (3)°, contributes to the general non-planarity of the mol­ecule. The phenol ring (C9–C14) is inclined by 45.73 (2)° to the aniline ring (C2–C7). The configuration of the C8=N1 bond of this Schiff base is *E*. The C14—O1 bond is 1.335 (5) Å, which is close to reported values of single C—O bonds in phenols and salicyl­idene­amines (Ozeryanskii *et al.*, 2006 ▸). The N1—C8 bond is short at 1.273 (4) Å, strongly indicating the existence of a conjugated C=N bond, while the longer C8—C9 bond [1.460 (5) Å] implies a single bond. All these data support the existence of the phenol–imine tautomer for (I) in its crystalline state. These features are similar to those observed in related 4-di­methyl­amino-*N*-salicylideneanilines (Pizzala *et al.*, 2000 ▸). The C—N, C=N and C—C bond lengths are normal and close to the values observed in related structures (Faizi *et al.*, 2017*a* ▸,*b* ▸).

## Supra­molecular features   

**Figure 2:**
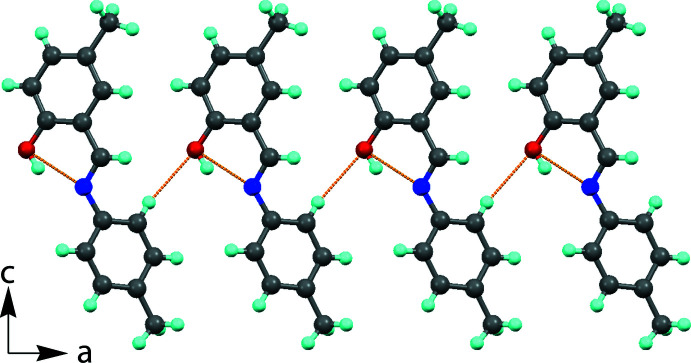
A view along the *b* axis of the polymeric chain formed *via* C—H⋯O inter­molecular hydrogen bonds (see Table 1 ▸).

In the crystal of (I), mol­ecules are linked by C—H⋯O inter­actions, forming sheets propagating along the *b*-axis direction (Fig. 2 ▸ and Table 1 ▸). There are no other significant inter­molecular inter­actions present.

## Hirshfeld surface analysis   

**Figure 3:**
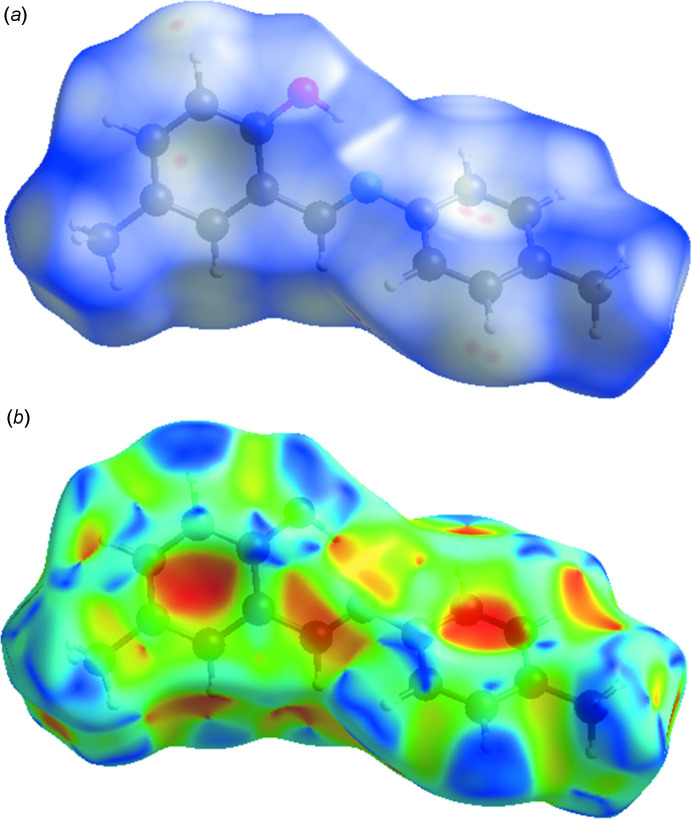
View of the three-dimensional Hirshfeld surfaces of (I) plotted over (*a*) *d*_norm_ and (*b*) shape-index.

In order to visualize the inter­molecular inter­actions in the crystal packing of (I), a Hirshfeld surface (HS) analysis (Hirshfeld, 1977 ▸; Spackman & Jayatilaka, 2009 ▸) was carried out using *Crystal Explorer 17.5* (Turner *et al.*, 2017 ▸). In the HS plotted over *d*_norm_ (Fig. 3 ▸ *a*), white indicates contacts with distances equal to the sum of van der Waals radii, while the red and blue colours indicate distances shorter (in close contact) or longer (distinct contact) than the van der Waals radii, respectively (Venkatesan *et al.*, 2016 ▸). The bright-red spots indicate their roles as respective donors and/or acceptors. The shape-index of the HS is a tool to visualize *π*–*π* stacking by the presence of adjacent red and blue triangles; if there are no adjacent red and/or blue triangles, then there are no *π*–*π* inter­actions. Fig. 3 ▸ *b* clearly suggests that there are no *π*–*π* inter­actions in (I).

**Figure 4:**
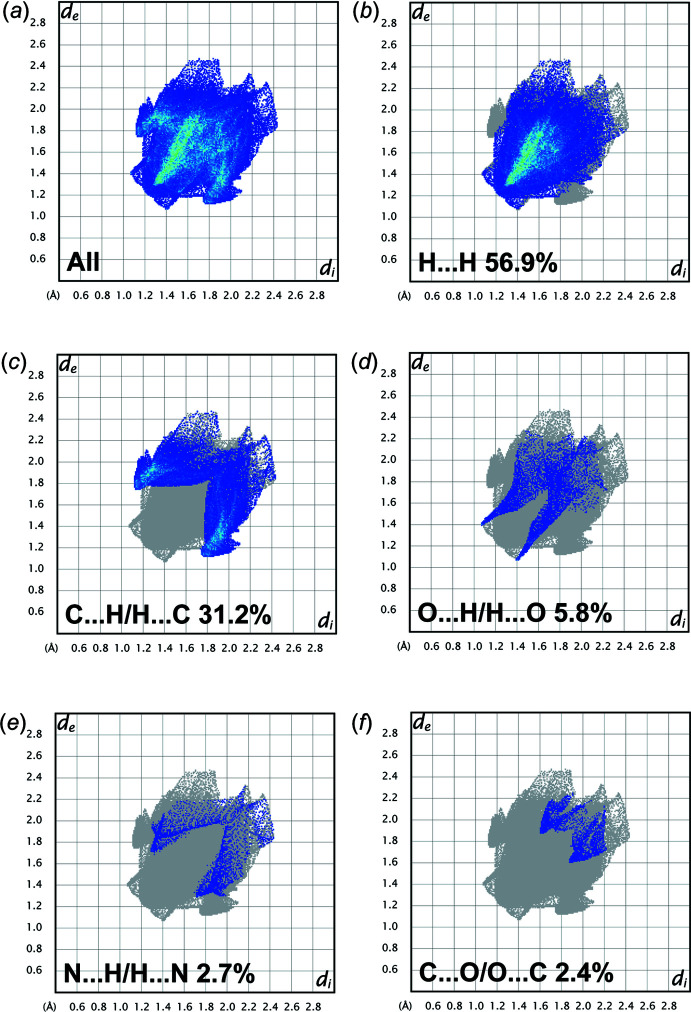
(*a*) Full two-dimensional fingerprint plot of (I), and those delineated into (*b*) H⋯H, (*c*) H⋯C/C⋯H, (*d*) H⋯O/O⋯H, (*e*) H⋯N/N⋯H and (*f*) C⋯O/O⋯C inter­actions.

The overall two-dimensional fingerprint plot (McKinnon *et al.*, 2007 ▸) is shown in Fig. 4 ▸ *a*, and those delineated into H⋯H, H⋯C/C⋯H, H⋯O/O⋯H, H⋯N/N⋯H and C⋯O/O⋯C contacts are illustrated in Fig. 4 ▸ *b*–*f*, respectively. The most important inter­action is H⋯H, contributing to 56.9% to the overall crystal packing (Fig. 4 ▸ *b*). The fingerprint plot delin­eated into H⋯C/C⋯H contacts (31.2% contribution to the HS) shows a pair of characteristic wings, Fig. 4 ▸ *c*. The scattered points in a pair of spikes are seen in the fingerprint plot for H⋯O/O⋯H contacts (Fig. 4 ▸ *d*, 5.8% contribution). H⋯N/N⋯H contacts contribute 2.7% (Fig. 4 ▸ *e*). The scattered points form a the pair of spikes in the fingerprint plot delineated into C⋯O/O⋯C contacts (Fig. 4 ▸ *f*, 2.4% contribution). The other inter­actions are C⋯C (0.8%) and O⋯N/C⋯N (0.1%). The Hirshfeld surface analysis confirms the importance of H-atom contacts in establishing the packing. The large number of H⋯H and H⋯C/C⋯H inter­actions suggest that van der Waals inter­actions and hydrogen bonding play the main roles in the crystal packing (Hathwar *et al.*, 2015 ▸).

## DFT calculations   

**Figure 5:**
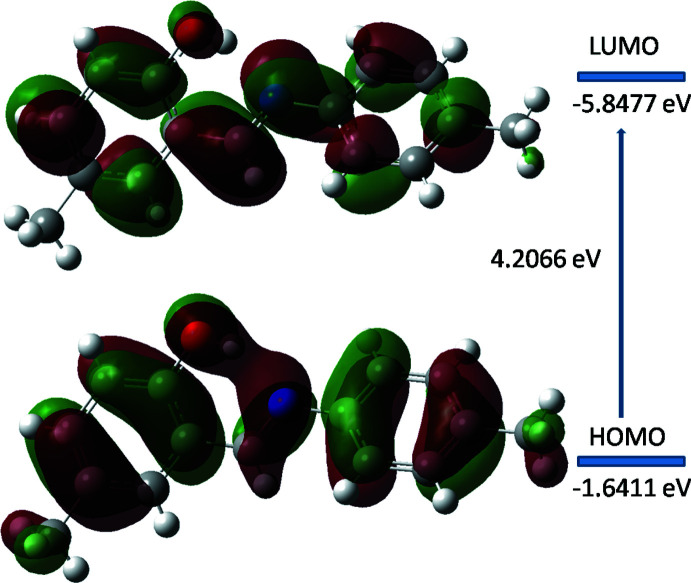
Energy band gap of the title compound (I).

The optimized structure in the gas phase of compound (I) was generated theoretically *via* density functional theory (DFT) using the standard B3LYP functional and 6–311 G(d,p) basis-set calculations (Becke, 1993 ▸) as implemented in *GAUSSIAN 09* (Frisch *et al.*, 2009 ▸). The theoretical and experimental results are in good agreement (Table 2 ▸). The highest-occupied mol­ecular orbital (HOMO), acting as an electron donor, and the lowest-unoccupied mol­ecular orbital (LUMO), acting as an electron acceptor, are very important parameters for quantum chemistry. When the energy gap is small, the mol­ecule is highly polarizable and has high chemical reactivity (Fukui, 1982 ▸; Khan *et al.*, 2015 ▸). The DFT calculations provide some important information on the reactivity and site selectivity of the mol­ecular framework, *E*_HOMO_ and *E*_LUMO_, which clarify the inevitable charge-exchange collaboration inside the studied material. These data, which also include the electronegativity (χ), hardness (η), electrophilicity (ω), softness (*σ*) and fraction of electrons transferred (*ΔN*) are recorded in Table 3 ▸. The significance of η and *σ* is for the evaluation of both the reactivity and stability. The electron transition from the HOMO to the LUMO energy level is shown in Fig. 5 ▸. The HOMO and LUMO are localized in the plane extending from the whole phenol ring. The energy band gap [Δ*E* = *E*_LUMO_-*E*_HOMO_] of the mol­ecule is 2.742 eV, the frontier mol­ecular orbital energies *E*_HOMO_ and *E*_LUMO_ being −1.6411eV and −5.8477 eV, respectively. The dipole moment of (I) is estimated to be 2.61 Debye.

## Database survey   

A search of the Cambridge Structural Database (CSD, version 5.39; Groom *et al.*, 2016 ▸) gave six hits for the {[(3-hy­droxy­phen­yl)imino]­meth­yl}phenol moiety. Two compounds that are very similar compound to (I) have been reported in the literature, *viz. N*-(3-hy­droxy­phen­yl)-5-meth­oxy­salicylaldimine (BALHUS; Popović *et al.*, 2002 ▸) in which a meth­oxy group replaces the methyl group and 4-chloro-2-{[(3-hy­droxy­phen­yl)imino]­meth­yl}phenol (ISENIE; Yu *et al.*, 2011 ▸) in which the methyl group is replaced by a chloro group. In the cobalt and manganese complexes di­aqua-bis­{2-hy­droxy-4-[(2-hy­droxy­benzyl­idene)amino]­benzoato-*O*}bis­(methanol)cobalt(II) (SUL­HOX; Zhou *et al.* 2009 ▸) and (2,2′-{ethane-1,2-diylbis[(nitrilo)­methylyl­idene]}diphenolato){2-hy­droxy-4-[(2-hy­drox­y­benzyl­idene)amino]­benzoato}manganese(III) (UQUBEO; Chen *et al.*, 2011 ▸), the methyl group of (I) is replaced by an ester and acts as a ligand. A similar compound, 2-hy­droxy-*N*′-(2-hy­droxy­benzyl­idene)-4-[(2-hy­droxy­benzyl­idene)amino]­benzohydrazide (TAXRUI; Mitra *et al.*, 2017 ▸) is substituted at the methyl group of (I). All these compounds have a similar intra­molecular O—H⋯N hydrogen bond present, forming an *S*(6) ring motif.

## Synthesis and crystallization   

The title compound (I) was obtained following a published method (Hanika *et al.*, 1971 ▸; Samant & Mayadeo 1982 ▸). Single crystals of compound (I) were obtained by slow evaporation of an ethanol solution after 4 d.

## Refinement   

Crystal data, data collection and structure refinement details are summarized in Table 4 ▸. All H atoms were placed in geometrically idealized positions and constrained to ride on their parent atoms, with C—H = 0.93–0.96 Å and *U*_iso_(H) = 1.*2U*_eq_ or 1.*5U*_eq_(C,O). The crystal studied was refined an a perfect inversion twin.

## Supplementary Material

Crystal structure: contains datablock(s) I. DOI: 10.1107/S2056989020007847/tx2025sup1.cif

Structure factors: contains datablock(s) I. DOI: 10.1107/S2056989020007847/tx2025Isup2.hkl

Supporting information file. DOI: 10.1107/S2056989020007847/tx2025Isup3.cml

CCDC reference: 2009052

Additional supporting information:  crystallographic information; 3D view; checkCIF report

## Figures and Tables

**Table 1:** Hydrogen-bond geometry (Å, °)

*D*—H⋯*A*	*D*—H	H⋯*A*	*D*⋯*A*	*D*—H⋯*A*
O1—H1⋯N1	0.82	1.87	2.591 (4)	146
C4—H4⋯O1^i^	0.93	2.60	3.448 (5)	152

Symmetry code: (i) 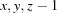.

**Table 2:** Comparison of observed (X-ray data) and calculated (DFT) geometric parameters (Å, °)

Parameter	X-ray	B3LYP/6–311G(d,p)
O1—C14	1.335 (5)	1.335
N1—C8	1.273 (4)	1.273
N1—C5	1.419 (5)	1.419
C1—C2	1.499 (5)	1.499
C11—C15	1.521 (6)	1.521
C8—C9	1.460 (5)	1.460
		
C8—N1—C5	120.6 (3)	120.6
N1—C8—C9	120.6 (3)	120.6
		
C5—N1—C8—C9	−169.8 (3)	−169.8

**Table 3:** Calculated mol­ecular energies for (I)

Mol­ecular Energy (a.u.) (eV)	Compound (I)
Total Energy, *TE* (eV)	−19333.931
*E*_HOMO_ (eV)	−1.641
*E*_LUMO_ (eV)	−5.848
Gap, *ΔE* (eV)	4.207
Dipole moment, *μ* (Debye)	2.61
Ionization potential, *I* (eV)	1.641
Electron affinity, *A*	5.848
Electronegativity, *χ*	3.744
Hardness, *η*	2.103
Electrophilicity index, *ω*	3.333
Softness, *σ*	0.238
Fraction of electron transferred, *ΔN*	0.774

**Table 4:** Experimental details

Crystal data	
Chemical formula	C_15_H_15_NO
*M*_r_	225.28
Crystal system, space group	Monoclinic, *P* *c*
Temperature (K)	296
*a*, *b*, *c* (Å)	13.8433 (10), 7.0774 (6), 6.2142 (5)
β (°)	95.517 (6)
*V* (Å^3^)	606.01 (8)
*Z*	2
Radiation type	Mo *K*α
μ (mm^−1^)	0.08
Crystal size (mm)	0.75 × 0.53 × 0.14

Data collection	
Diffractometer	Stoe IPDS 2
Absorption correction	Integration (*X-RED32*; Stoe & Cie, 2002 ▸)
*T*_min_, *T*_max_	0.944, 0.989
No. of measured, independent and observed [*I* > 2σ(*I*)] reflections	9856, 4081, 2430
*R*_int_	0.063

Refinement	
*R*[*F* ^2^ > 2σ(*F* ^2^)], *wR*(*F* ^2^), *S*	0.068, 0.199, 1.05
No. of reflections	4081
No. of parameters	154
No. of restraints	2
H-atom treatment	H-atom parameters constrained
Δρ_max_, Δρ_min_ (e Å^−3^)	0.22, −0.18
Absolute structure	Refined as an inversion twin
Absolute structure parameter	0.5

Computer programs: *X-AREA* and *X-RED32* (Stoe & Cie, 2002 ▸), *SHELXT2018/3* (Sheldrick, 2015*a* ▸), *SHELXL2018/3* (Sheldrick, 2015*b* ▸), *OLEX2* (Dolomanov *et al.*, 2009 ▸), *Mercury* (Macrae *et al.*, 2020 ▸), *WinGX* (Farrugia, 2012 ▸), *PLATON* (Spek, 2020 ▸) and *publCIF* (Westrip, 2010 ▸).

## supplementary crystallographic information

### Crystal data

**Table:**

C_15_H_15_NO	*F*(000) = 240
*M**_r_* = 225.28	*D*_x_ = 1.235 Mg m^−^^3^
Monoclinic, *P**c*	Mo *K*α radiation, λ = 0.71073 Å
*a* = 13.8433 (10) Å	Cell parameters from 12502 reflections
*b* = 7.0774 (6) Å	θ = 2.9–32.2°
*c* = 6.2142 (5) Å	µ = 0.08 mm^−^^1^
β = 95.517 (6)°	*T* = 296 K
*V* = 606.01 (8) Å^3^	Prism, yellow
*Z* = 2	0.75 × 0.53 × 0.14 mm

### Data collection

**Table:**

STOE IPDS 2 diffractometer	4081 independent reflections
Radiation source: sealed X-ray tube, 12 x 0.4 mm long-fine focus	2430 reflections with *I* > 2σ(*I*)
Plane graphite monochromator	*R*_int_ = 0.063
Detector resolution: 6.67 pixels mm^-1^	θ_max_ = 31.9°, θ_min_ = 3.0°
rotation method scans	*h* = −20→20
Absorption correction: integration (X-RED32; Stoe & Cie, 2002)	*k* = −10→10
*T*_min_ = 0.944, *T*_max_ = 0.989	*l* = −9→9
9856 measured reflections	

### Refinement

**Table:**

Refinement on *F*^2^	Secondary atom site location: difference Fourier map
Least-squares matrix: full	Hydrogen site location: inferred from neighbouring sites
*R*[*F*^2^ > 2σ(*F*^2^)] = 0.068	H-atom parameters constrained
*wR*(*F*^2^) = 0.199	*w* = 1/[σ^2^(*F*_o_^2^) + (0.0974*P*)^2^ + 0.0159*P*] where *P* = (*F*_o_^2^ + 2*F*_c_^2^)/3
*S* = 1.05	(Δ/σ)_max_ < 0.001
4081 reflections	Δρ_max_ = 0.22 e Å^−^^3^
154 parameters	Δρ_min_ = −0.18 e Å^−^^3^
2 restraints	Absolute structure: Refined as an inversion twin
Primary atom site location: structure-invariant direct methods	Absolute structure parameter: 0.5

### Special details

**Table:**

Geometry. All esds (except the esd in the dihedral angle between two l.s. planes) are estimated using the full covariance matrix. The cell esds are taken into account individually in the estimation of esds in distances, angles and torsion angles; correlations between esds in cell parameters are only used when they are defined by crystal symmetry. An approximate (isotropic) treatment of cell esds is used for estimating esds involving l.s. planes.

### Fractional atomic coordinates and isotropic or equivalent isotropic displacement parameters (Å^2^)

**Table:**

	*x*	*y*	*z*	*U*_iso_*/*U*_eq_	
O1	1.5639 (2)	1.6821 (5)	1.9220 (4)	0.0603 (8)	
H1	1.515349	1.711085	1.842779	0.090*	
C14	1.6429 (3)	1.7148 (5)	1.8202 (5)	0.0440 (8)	
N1	1.4649 (2)	1.7758 (4)	1.5628 (5)	0.0469 (8)	
C7	1.2034 (3)	1.8103 (6)	1.3960 (8)	0.0518 (10)	
H7	1.148245	1.853517	1.454950	0.062*	
C5	1.3754 (3)	1.7675 (5)	1.4311 (6)	0.0429 (8)	
C10	1.7225 (3)	1.8176 (5)	1.5071 (6)	0.0436 (7)	
H10	1.718715	1.864633	1.366824	0.052*	
C8	1.5439 (2)	1.8060 (6)	1.4792 (5)	0.0425 (8)	
H8	1.542131	1.842677	1.335168	0.051*	
C9	1.6373 (3)	1.7836 (5)	1.6074 (6)	0.0424 (8)	
C3	1.2783 (3)	1.6698 (6)	1.1093 (7)	0.0511 (9)	
H3	1.274388	1.616264	0.972030	0.061*	
C6	1.2917 (3)	1.8269 (6)	1.5136 (6)	0.0510 (10)	
H6	1.295318	1.878997	1.651540	0.061*	
C2	1.1939 (3)	1.7306 (6)	1.1904 (6)	0.0507 (10)	
C13	1.7325 (3)	1.6749 (6)	1.9267 (6)	0.0522 (10)	
H13	1.737103	1.626082	2.066301	0.063*	
C4	1.3679 (3)	1.6860 (5)	1.2251 (6)	0.0475 (8)	
H4	1.423221	1.642909	1.166639	0.057*	
C12	1.8156 (3)	1.7078 (6)	1.8251 (7)	0.0537 (11)	
H12	1.875444	1.679660	1.899117	0.064*	
C15	1.9044 (3)	1.8119 (7)	1.5057 (9)	0.0700 (12)	
H15A	1.886555	1.863232	1.364448	0.105*	
H15B	1.946511	1.898674	1.587855	0.105*	
H15C	1.937339	1.693734	1.492355	0.105*	
C11	1.8135 (3)	1.7801 (5)	1.6204 (7)	0.0485 (9)	
C1	1.0972 (3)	1.7067 (8)	1.0630 (9)	0.0758 (15)	
H1A	1.047278	1.756598	1.143908	0.114*	
H1B	1.096915	1.773211	0.928331	0.114*	
H1C	1.085349	1.574848	1.035150	0.114*	

### Atomic displacement parameters (Å^2^)

**Table:**

	*U*^11^	*U*^22^	*U*^33^	*U*^12^	*U*^13^	*U*^23^
O1	0.0522 (14)	0.087 (2)	0.0425 (13)	−0.0019 (15)	0.0088 (11)	0.0087 (15)
C14	0.052 (2)	0.0442 (19)	0.0359 (17)	−0.0002 (15)	0.0035 (15)	0.0015 (15)
N1	0.0464 (16)	0.0464 (18)	0.0479 (19)	−0.0010 (14)	0.0045 (13)	0.0029 (14)
C7	0.0372 (18)	0.054 (2)	0.065 (3)	0.0014 (16)	0.0097 (17)	−0.007 (2)
C5	0.0459 (18)	0.0420 (18)	0.0406 (19)	−0.0030 (16)	0.0037 (15)	0.0054 (16)
C10	0.0442 (16)	0.0428 (17)	0.0435 (18)	−0.0017 (15)	0.0030 (14)	0.0031 (15)
C8	0.0434 (18)	0.0474 (19)	0.0359 (17)	−0.0036 (14)	−0.0001 (14)	0.0054 (14)
C9	0.0421 (16)	0.0426 (18)	0.042 (2)	−0.0034 (15)	0.0014 (15)	−0.0044 (16)
C3	0.054 (2)	0.051 (2)	0.048 (2)	−0.0009 (18)	−0.0012 (17)	−0.0079 (18)
C6	0.057 (2)	0.050 (2)	0.048 (2)	0.0011 (18)	0.0102 (18)	0.0016 (18)
C2	0.046 (2)	0.045 (2)	0.060 (2)	0.0003 (16)	0.0012 (18)	0.0048 (18)
C13	0.059 (2)	0.0455 (19)	0.050 (2)	−0.0028 (18)	−0.0029 (19)	−0.0017 (17)
C4	0.0477 (19)	0.0468 (19)	0.050 (2)	0.0037 (15)	0.0117 (15)	0.0010 (16)
C12	0.045 (2)	0.052 (3)	0.061 (3)	0.0010 (15)	−0.0061 (18)	−0.0008 (18)
C15	0.051 (2)	0.078 (3)	0.082 (3)	−0.001 (2)	0.010 (2)	0.007 (3)
C11	0.0428 (18)	0.0413 (18)	0.062 (2)	−0.0055 (14)	0.0066 (17)	−0.0073 (16)
C1	0.046 (2)	0.081 (3)	0.096 (4)	−0.002 (2)	−0.014 (2)	−0.002 (3)

### Geometric parameters (Å, º)

**Table:**

O1—C14	1.335 (5)	C3—C2	1.384 (6)
O1—H1	0.8200	C3—H3	0.9300
C14—C13	1.378 (5)	C6—H6	0.9300
C14—C9	1.404 (5)	C2—C1	1.499 (5)
N1—C8	1.273 (4)	C13—C12	1.384 (6)
N1—C5	1.419 (5)	C13—H13	0.9300
C7—C6	1.368 (6)	C4—H4	0.9300
C7—C2	1.391 (6)	C12—C11	1.369 (6)
C7—H7	0.9300	C12—H12	0.9300
C5—C6	1.376 (5)	C15—C11	1.521 (6)
C5—C4	1.399 (6)	C15—H15A	0.9600
C10—C9	1.407 (5)	C15—H15B	0.9600
C10—C11	1.408 (5)	C15—H15C	0.9600
C10—H10	0.9300	C1—H1A	0.9600
C8—C9	1.460 (5)	C1—H1B	0.9600
C8—H8	0.9300	C1—H1C	0.9600
C3—C4	1.378 (5)		
			
C14—O1—H1	109.5	C3—C2—C1	121.0 (4)
O1—C14—C13	118.5 (3)	C7—C2—C1	122.1 (4)
O1—C14—C9	122.2 (3)	C14—C13—C12	119.7 (4)
C13—C14—C9	119.3 (3)	C14—C13—H13	120.2
C8—N1—C5	120.6 (3)	C12—C13—H13	120.2
C6—C7—C2	121.7 (4)	C3—C4—C5	119.7 (4)
C6—C7—H7	119.1	C3—C4—H4	120.1
C2—C7—H7	119.1	C5—C4—H4	120.1
C6—C5—C4	118.4 (4)	C11—C12—C13	122.9 (3)
C6—C5—N1	119.5 (3)	C11—C12—H12	118.6
C4—C5—N1	121.9 (3)	C13—C12—H12	118.6
C9—C10—C11	119.6 (4)	C11—C15—H15A	109.5
C9—C10—H10	120.2	C11—C15—H15B	109.5
C11—C10—H10	120.2	H15A—C15—H15B	109.5
N1—C8—C9	120.6 (3)	C11—C15—H15C	109.5
N1—C8—H8	119.7	H15A—C15—H15C	109.5
C9—C8—H8	119.7	H15B—C15—H15C	109.5
C14—C9—C10	120.2 (3)	C12—C11—C10	118.2 (4)
C14—C9—C8	121.2 (3)	C12—C11—C15	123.1 (3)
C10—C9—C8	118.4 (3)	C10—C11—C15	118.6 (4)
C4—C3—C2	122.2 (4)	C2—C1—H1A	109.5
C4—C3—H3	118.9	C2—C1—H1B	109.5
C2—C3—H3	118.9	H1A—C1—H1B	109.5
C7—C6—C5	121.0 (4)	C2—C1—H1C	109.5
C7—C6—H6	119.5	H1A—C1—H1C	109.5
C5—C6—H6	119.5	H1B—C1—H1C	109.5
C3—C2—C7	116.9 (3)		
			
C8—N1—C5—C6	−148.2 (4)	C4—C3—C2—C7	0.2 (6)
C8—N1—C5—C4	37.2 (6)	C4—C3—C2—C1	−178.4 (4)
C5—N1—C8—C9	−169.8 (3)	C6—C7—C2—C3	−0.5 (6)
O1—C14—C9—C10	179.4 (4)	C6—C7—C2—C1	178.1 (4)
C13—C14—C9—C10	−2.2 (5)	O1—C14—C13—C12	−179.9 (3)
O1—C14—C9—C8	−5.9 (5)	C9—C14—C13—C12	1.7 (6)
C13—C14—C9—C8	172.5 (4)	C2—C3—C4—C5	−0.5 (6)
C11—C10—C9—C14	0.8 (5)	C6—C5—C4—C3	1.0 (6)
C11—C10—C9—C8	−174.0 (3)	N1—C5—C4—C3	175.6 (4)
N1—C8—C9—C14	4.9 (6)	C14—C13—C12—C11	0.2 (6)
N1—C8—C9—C10	179.6 (3)	C13—C12—C11—C10	−1.7 (6)
C2—C7—C6—C5	1.0 (7)	C13—C12—C11—C15	−178.4 (4)
C4—C5—C6—C7	−1.3 (6)	C9—C10—C11—C12	1.1 (5)
N1—C5—C6—C7	−176.0 (4)	C9—C10—C11—C15	178.0 (3)

### Hydrogen-bond geometry (Å, º)

**Table:**

*D*—H···*A*	*D*—H	H···*A*	*D*···*A*	*D*—H···*A*
O1—H1···N1	0.82	1.87	2.591 (4)	146
C4—H4···O1^i^	0.93	2.60	3.448 (5)	152

Symmetry code: (i) *x*, *y*, *z*−1.

## References

[bb1] Becke, A. D. (1993). *J. Chem. Phys.* **98**, 5648–5652.

[bb2] Chen, H., Zhou, R.-W., Ma, C.-B., Hu, M.-Q. & Chen, C.-N. (2011). *Chin. J. Struct. Chem.* **30**, 158–163.

[bb3] Dalapati, S., Alam, M. A., Jana, S. & Guchhait, N. (2011). *J. Fluor. Chem.* **132**, 536–540.

[bb4] Dolomanov, O. V., Bourhis, L. J., Gildea, R. J., Howard, J. A. K. & Puschmann, H. (2009). *J. Appl. Cryst.* **42**, 339–341.

[bb5] Elmalı, A., Kabak, M., Kavlakoğlu, E., Elerman, Y. & Durlu, T. N. (1999). *J. Mol. Struct.* **510**, 207–214.

[bb6] El-masry, A. H., Fahmy, H. H. & Ali Abdelwahed, S. (2000). *Molecules*, **5**, 1429–1438.

[bb7] Faizi, M. S. H., Ahmad, M., Kapshuk, A. A. & Golenya, I. A. (2017*a*). *Acta Cryst.* E**73**, 38–40. 10.1107/S2056989016019733 PMC5209767 28083131

[bb8] Faizi, M. S. H., Alam, M. J., Haque, A., Ahmad, S., Shahid, M. & Ahmad, M. (2018). *J. Mol. Struct.* **1156**, 457–464.

[bb9] Faizi, M. S. H., Ali, A. & Potaskalov, V. A. (2016). *Acta Cryst.* E**72**, 1366–1369. 10.1107/S205698901601344X PMC5050754 27746919

[bb10] Faizi, M. S. H., Dege, N., Haque, A., Kalibabchuk, V. A. & Cemberci, M. (2017*b*). *Acta Cryst.* E**73**, 96–98. 10.1107/S2056989016020107 PMC5290541 28217318

[bb11] Farrugia, L. J. (2012). *J. Appl. Cryst.* **45**, 849–854.

[bb12] Frisch, M. J., Trucks, G. W., Schlegel, H. B., Scuseria, G. E., Robb, M. A., Cheeseman, J. R., Scalmani, G., Barone, V., Mennucci, B., Petersson, G. A., Nakatsuji, H., Caricato, M., Li, X., Hratchian, H. P., Izmaylov, A. F., Bloino, J., Zheng, G., Sonnenberg, J. L., Hada, M., Ehara, M., Toyota, K., Fukuda, R., Hasegawa, J., Ishida, M., Nakajima, T., Honda, Y., Kitao, O., Nakai, H., Vreven, T., Montgomery, J. A. Jr, Peralta, J. E., Ogliaro, F., Bearpark, M., Heyd, J. J., Brothers, E., Kudin, K. N., Staroverov, V. N., Kobayashi, R., Normand, J., Raghavachari, K., Rendell, A., Burant, J. C., Iyengar, S. S., Tomasi, J., Cossi, M., Rega, N., Millam, J. M., Klene, M., Knox, J. E., Cross, J. B., Bakken, V., Adamo, C., Jaramillo, J., Gomperts, R., Stratmann, R. E., Yazyev, O., Austin, A. J., Cammi, R., Pomelli, C., Ochterski, J. W., Martin, R. L., Morokuma, K., Zakrzewski, V. G., Voth, G. A., Salvador, P., Dannenberg, J. J., Dapprich, S., Daniels, A. D., Farkas, Ö., Foresman, J. B., Ortiz, J. V., Cioslowski, J. & Fox, D. J. (2009). *GAUSSIAN09*. Gaussian Inc., Wallingford, CT, USA.

[bb13] Fukui, K. (1982). *Science*, **218**, 747–754. 10.1126/science.218.4574.747 17771019

[bb14] Groom, C. R., Bruno, I. J., Lightfoot, M. P. & Ward, S. C. (2016). *Acta Cryst.* B**72**, 171–179. 10.1107/S2052520616003954 PMC4822653 27048719

[bb15] Hanika, J., Sporka, K. & Ruzicka, V. (1971). *Chem. Commun.* **36**, 3608–3620.

[bb16] Hathwar, V. R., Sist, M., Jørgensen, M. R. V., Mamakhel, A. H., Wang, X., Hoffmann, C. M., Sugimoto, K., Overgaard, J. & Iversen, B. B. (2015). *IUCrJ*, **2**, 563–574. 10.1107/S2052252515012130 PMC4547824 26306198

[bb17] Hirshfeld, H. L. (1977). *Theor. Chim. Acta*, **44**, 129–138.

[bb18] Hökelek, T., Bilge, S., Demiriz, Ş., Özgüç, B. & Kılıç, Z. (2004). *Acta Cryst.* C**60**, o803–o805. 10.1107/S0108270104022565 15528825

[bb19] Iwan, A., Kaczmarczyk, B., Janeczek, H., Sek, D. & Ostrowski, S. (2007). *Spectrochim. Acta A Mol. Biomol. Spectrosc.* **66**, 1030–1041. 10.1016/j.saa.2006.05.016 16872877

[bb20] Kansız, S. & Dege, N. (2018). *J. Mol. Struct.* **1173**, 42–51.

[bb21] Khan, E., Shukla, A., Srivastava, A., Shweta, P. & Tandon, P. (2015). *New J. Chem.* **39**, 9800–9812.

[bb22] Kumar, M., Kumar, A., Faizi, M. S. H., Kumar, S., Singh, M. K., Sahu, S. K., Kishor, S. & John, R. P. (2018). *Sens. Actuators B Chem.* **260**, 888–899.

[bb23] Macrae, C. F., Sovago, I., Cottrell, S. J., Galek, P. T. A., McCabe, P., Pidcock, E., Platings, M., Shields, G. P., Stevens, J. S., Towler, M. & Wood, P. A. (2020). *J. Appl. Cryst.* **53**, 226–235. 10.1107/S1600576719014092 PMC6998782 32047413

[bb24] McKinnon, J. J., Jayatilaka, D. & Spackman, M. A. (2007). *Chem. Commun.* pp. 3814–3816. 10.1039/b704980c 18217656

[bb25] Mitra, S., Sasmal, H. S., Kundu, T., Kandambeth, S., Illath, K., Díaz Díaz, D. & Banerjee, R. (2017). *J. Am. Chem. Soc.* **139**, 4513–4520. 10.1021/jacs.7b00925 28256830

[bb26] Moroz, Y. S., Demeshko, S., Haukka, M., Mokhir, A., Mitra, U., Stocker, M., Müller, P., Meyer, F. & Fritsky, I. O. (2012). *Inorg. Chem.* **51**, 7445–7447. 10.1021/ic300902z 22765646

[bb27] Mukherjee, P., Das, A., Faizi, M. S. H. & Sen, P. (2018). *Chemistry Select*, **3**, 3787–3796.

[bb28] Ozeryanskii, V. A., Pozharskii, A. F., Schilf, W., Kamieński, B., Sawka-Dobrowolska, W., Sobczyk, L. & Grech, E. (2006). *Eur. J. Org. Chem.* pp. 782–790.

[bb29] Pizzala, H., Carles, M., Stone, W. E. E. & Thevand, A. (2000). *J. Chem. Soc. Perkin Trans. 2*, pp. 935–939.

[bb30] Popović, Z., Pavlović, G., Matković-Čalogović, D., Roje, V. & Leban, I. (2002). *J. Mol. Struct.* **615**, 23–31.

[bb31] Samant, S. D. & Mayadeo, M. S. (1982). *J. Indian Chem. Soc.* **14**, 383–384.

[bb32] Sheldrick, G. M. (2015*a*). *Acta Cryst.* A**71**, 3–8.

[bb33] Sheldrick, G. M. (2015*b*). *Acta Cryst.* C**71**, 3–8.

[bb34] Spackman, M. A. & Jayatilaka, D. (2009). *CrystEngComm*, **11**, 19–32.

[bb35] Spek, A. L. (2020). *Acta Cryst.* E**76**, 1–11. 10.1107/S2056989019016244 PMC6944088 31921444

[bb36] Stoe & Cie (2002). *X-AREA* and *X-RED32*. Stoe & Cie GmbH, Darmstadt, Germany.

[bb37] Sun, Y., Wang, Y., Liu, Z., Huang, C. & Yu, C. (2012). *Spectrochim. Acta A Mol. Biomol. Spectrosc.* **96**, 42–50. 10.1016/j.saa.2012.04.094 22652542

[bb38] Turner, M. J., Mckinnon, J. J., Wolff, S. K., Grimwood, D. J., Spackman, P. R., Jayatilaka, D. & Spackman, M. A. (2017). *Crystal Explorer 17.5*. The University of Western Australia.

[bb39] Venkatesan, P., Thamotharan, S., Ilangovan, A., Liang, H. & Sundius, T. (2016). *Spectrochim. Acta A Mol. Biomol. Spectrosc.* **153**, 625–636. 10.1016/j.saa.2015.09.002 26452098

[bb40] Westrip, S. P. (2010). *J. Appl. Cryst.* **43**, 920–925.

[bb41] Yu, J., Huang, D., Hong, Y. & Huang, K. (2011). *Z. Kristallogr. New Cryst. Struct.* **226**, 275–276.

[bb42] Zhou, R.-W., Ma, C.-B., Wang, M., Chen, H. & Chen, C.-N. (2009). *Chin. J. Struct. Chem.* **28**, 864–868.

